# Platinum-Resistant Ovarian Cancer Is Vulnerable to the cJUN-XRCC4 Pathway Inhibition

**DOI:** 10.3390/cancers14246068

**Published:** 2022-12-09

**Authors:** Manman Xu, Xi Huang, Cuimiao Zheng, Junming Long, Qingyuan Dai, Yangyang Chen, Jingyi Lu, Chaoyun Pan, Shuzhong Yao, Jie Li

**Affiliations:** 1 Department of Obstetrics and Gynecology, The First Affiliated Hospital, Sun Yat-sen University, Guangzhou 510080, China; 2 Department of Biochemistry and Molecular Biology, Zhongshan School of Medicine, Sun Yat-sen University, Guangzhou 510080, China; 3 Advanced Medical Technology Center, The First Affiliated Hospital, Sun Yat-sen University, Guangzhou 510080, China

**Keywords:** ovarian cancer, platinum resistance, DNA repair

## Abstract

**Simple Summary:**

Although the initial clinical response of ovarian cancer patients to first-line platinum-based chemotherapy is often excellent, most patients relapse and develop resistance to treatment. However, the mechanism underlying resistance is unclear. DNA repair is the best-known effector of resistance to platinum-based agents, which damages DNA and activates DNA damage response. Two major DNA repair pathways exist, including homologous recombination (HR) and nonhomologous end joining (NHEJ). While the role of HR in platinum resistance is well studied, how NHEJ machinery affects platinum resistance in ovarian cancer remains largely unexplored. The goal of the current study is to decipher how NHEJ is involved in platinum resistance in ovarian cancer. Our study demonstrates XRCC4 in the NHEJ pathway specifically contributes to platinum resistance by mitigating the DNA damage caused by platinum drugs and provides preclinical evidence for targeting XRCC4 as a new strategy to battle cisplatin resistance in ovarian cancer treatment.

**Abstract:**

DNA double-strand breaks (DSBs) caused by platinum drugs are dangerous lesions that kill cancer cells in chemotherapy. Repair of DSB by homologous recombination (HR) and nonhomologous end joining (NHEJ) is frequently associated with platinum resistance in ovarian cancer. While the role of the HR pathway and HR-targeting strategy in platinum resistance is well studied, dissecting and targeting NHEJ machinery to overcome platinum resistance in ovarian cancer remain largely unexplored. Here, through an NHEJ pathway-focused gene RNAi screen, we found that the knockdown of XRCC4 significantly sensitized cisplatin treatment in the platinum-resistant ovarian cancer cell lines. Moreover, upregulation of XRCC4 is observed in a panel of platinum-resistant cell lines relative to the parental cell lines, as well as in ovarian cancer patients with poor progression-free survival. Mechanistically, the increased sensitivity to cisplatin upon XRCC4 knockdown was caused by accumulated DNA damage. In cisplatin-resistant ovarian cancer, the JNK-cJUN complex, activated by cisplatin, translocated into the nucleus and promoted the transcription of XRCC4 to confer cisplatin resistance. Knockdown of XRCC4 or treatment of the JNK inhibitor led to the attenuation of cisplatin-resistant tumor growth in the xenograft mouse models. These data suggest targeting XRCC4 is a potential strategy for ovarian cisplatin resistance in ovarian cancer.

## 1. Introduction

Ovarian cancer ranks fifth in cancer-associated deaths among women, accounting for more deaths than any other gynecologic cancer [1,2]. Platinum-based chemotherapy regimen has been the most actively used as a first-line treatment of ovarian cancer for decades [3]. While highly effective at the initial stage of treatment, platinum-based chemotherapy mostly encounters severe drug resistance in ovarian cancer patients at a later stage, which leads to tumor recurrence and patient death [4]. Understanding the fundamental mechanisms that drive platinum resistance will hold a direct clinical translation potential in the clinical treatment of ovarian cancer.

The platinum-based drugs cisplatin and carboplatin eradicate rapidly growing cancer cells by crosslinking and damaging DNA [5]. The damaged DNA, if unrepaired promptly, will trigger a DNA damage response (DDR) and activate cell apoptosis [6]. In the last decade, an increasing amount of studies have investigated platinum resistance in multiple cancers [7]. Ishida et al. reported the copper channel plays an important role in the cellular active transportation of cisplatin so that a copper chelator significantly enhanced tumor-specific uptake of cisplatin [8]. Pathways that support cell survival and redox balance have also been shown to promote cisplatin resistance in multiple cancers [9,10,11]. Liu et al. found that H2K79 methylation altered by C/EBPbeta increased platinum resistance in ovarian cancer, suggesting epigenetic regulation also significantly contributes to the development of platinum resistance [12]. Fundamentally, cancer cells that develop resistance to platinum-based therapy should be able to repair the damage caused by the drugs and thus return to the normal cell cycle. Indeed, the DNA damage response and repair pathways have been interrogated intensively to dissect the mechanism underlying platinum resistance [13]. Specifically, ovarian cancer cells with homologous recombination deficiency (HRD) are found to be highly sensitive to PAPR inhibition and platinum drugs [14]. Cancer cells with restored HR exhibit significant resistance to platinum drugs, underscoring the importance of HR in conferring cisplatin resistance [15,16]. However, while the HR pathway is highly recognized as a crucial factor in conferring platinum resistance, the nonhomologous end-joining (NHEJ) pathway, another parallel and critical pathway for DNA damage repair, is underappreciated.

NHEJ is not as accurate a DNA damage repair pathway as HR. HR uses the undamaged DNA template for the repair, and NHEJ quickly modifies and joins the broken DNA ends together with no regard for homology [17,18,19]. However, NHEJ is both quick and active during all stages of the cell cycle, but its efficiency is the highest during G2/M. HR is active primarily in the S-phase and has lower activity in G2/M. Presumably, NHEJ could be an important complementary pathway with HR to repair DNA damage in the whole cell cycle and to repair it in a time-saving manner at the expense of relatively low-fidelity [20,21,22,23]. Therefore, targeting the NHEJ pathway could hold significant clinical translation potential opportunities.

In this study, through an NHEJ pathway-focused gene RNAi screen, we identified XRCC4 as an important NHEJ factor to promote resistance to cisplatin in ovarian cancer. Knockdown of XRCC4 significantly sensitized cisplatin treatment in the platinum-resistant ovarian cancer cell lines. Moreover, upregulation of XRCC4 is observed in a panel of platinum-resistant cell lines relative to the parental cell lines, as well as in ovarian cancer patients with poor progression-free survival. We demonstrated that specific knockdown of XRCC4 significantly induced DNA damage in cisplatin-resistant ovarian cancer upon cisplatin treatment. Moreover, we found that cisplatin activated the JNK-cJUN complex, subsequently leading to the complex translocating into the nucleus and activating the transcription of XRCC4 to confer cisplatin resistance. We also demonstrated that knockdown of XRCC4 or treatment of the JNK inhibitor decreased cisplatin-resistant tumor growth in the xenograft mouse models, implying the clinical translation of targeting the cJUN-XRCC4 pathway in overcoming cisplatin resistance. The current study suggests targeting XCRR4 is a potential strategy for ovarian cisplatin resistance in ovarian cancer.

## 2. Materials and Methods

### 2.1. Cell Culture

Culture medium: A2780 and PEO1 cells (RPMI 1640 medium with 10% FBS); SKOV3 cells (McCoy’s 5a medium with 10% FBS); 293T and ES2 (Dulbecco Modified Eagle Medium with 10% FBS). PEO1, SKOV3, and ES2 were obtained from American Type Culture Collection. A2780 was purchased from Sigma-Aldrich (St. Louis, MO, USA). A2780 was derived from ovarian carcinoma, SKOV3 was derived from ovarian adenocarcinoma and was included in high-grade serous cancer cell lines, PEO1 was derived from serous adenocarcinoma, and ES2 was derived from clear cell carcinoma. All cell lines in the study were tested for mycoplasma contamination and authenticated by STR profiling. Cisplatin-resistant sublines of A2780, SKOV3, PEO1, and ES2 were generated by a serial passage of cells in the presence of increasing concentrations of cisplatin and maintained in the presence of 0.5 μg/mL cisplatin. Lentivirus production in 293T was described previously [24,25].

### 2.2. Antibodies and Small-Molecule Inhibitors

XRCC4 (Cat. No.: sc-271087) antibody was purchased from Santa Cruz Biotechnology Company (Dallas, TX, USA). Antibodies against phospho-Histone γH2AX S139 (Cat. No.: 9718), CHK2 (Cat. No.: 6334), phospho-CHK2 T68 (Cat. No.: 2197), ATM (Cat. No.: 2873), and phospho-ATM S1981 (Cat. No.: 5883) were purchased from Cell Signaling Technology (Danvers, MA, USA). The Alexa Fluor 488-conjugated anti-phospho-Histone γH2AX S139 (Ser139) antibody was purchased from Millipore (Burlington, MA, USA) (Cat. No.: 05-636-AF488). Antibodies against β-actin (Cat. No.: A1978/AC-15) and FLAG (Cat. No.: F1804/M2, F7425) were purchased from Sigma-Aldrich. SP600125 (Selleckchem; Cat. No.: S1460) was prepared as 10 mmol/L solutions in DMSO.

### 2.3. RNAi Screens

A customized lentiviral short-hairpin RNA (shRNA) library was used to perform the screen. The library was sorted out from the TRC Lentiviral shRNA Library. The gene list and related detailed target sequences are provided in Supplementary Table S1. Cells were seeded onto 96-well plates as 9 replicate for virus infection. Then, 48 h after the viral infection, DMSO, cisplatin (sublethal dose: 5 μg/mL A2780cisR and 2 μg/mL SKOV3cisR), or puromycin (0.5 μg/mL) was used to treat the cells in triplicates. CellTiter-Glo Luminescent Viability Assay (Promega) was applied to determine the cell viability after 2 days.

### 2.4. Cell Viability Assay and Colony Formation Assay

A total of 4000 cells/well were seeded onto 96-well plates one day before and then the indicated concentrations of drugs were used to treat the cells for 48 h. CellTiter-Glo Luminescent Viability Assay (Promega) was used to monitor the cell viability according to the manufacturer’s instructions. A total of 250 cells were seeded onto a 35 mm dish one day before for the colony-forming assay. The cells were then treated with PBS or cisplatin for 48 h and then maintained in a fresh complete medium for 10 more days for colony formation. Crystal violet (0.5%) was used to stain the colonies. The number of cell colonies was calculated by ImageJ software 1.53v.

### 2.5. Immunofluorescence

A 1 mL volume of cells at a concentration of 1 × 10^5^/mL was seeded onto the microscope cover glass in 12-well cell culture plates overnight. Then, cisplatin or PBS was used to treat the cells for 48 h. After the treatment, 4% paraformaldehyde was used to fix cells, which then were permeabilized with 0.5% Triton X-100 for 10 min, blocked with normal goat serum for 30 min, and incubated with Alexa Fluor 488-conjugated anti-phospho Histone H2A.X (Ser139) antibody (05-636-AF488; Millipore) for 1 h at room temperature. DAPI (1 mg/mL; Sigma-Aldrich) was used to counterstain the nuclei. Images were acquired using LEICA confocal microscopy.

### 2.6. Western Blot

The fresh cell culture or tissues were used to extract total cellular protein with the lysis buffer (50 mM Tris-HCl (pH 7.5), 150 mM NaCl, 1% NP-40, 0.5% Na-deoxycholate) containing the protease inhibitor cocktail (Roche, Indianapolis, IN, USA). A total of 30 μg protein was loaded for the 10% SDS-polyacrylamide gel electrophoresis, then transblotted onto nitrocellulose membrane (Biotanon, Shanghai, China). The membranes were incubated with the primary antibody at 4 °C overnight, then with HRP-conjugated secondary antibody for 1 h at room temperature. The specific blot band was detected with enhanced chemiluminescence. ImageJ software 1.53v was used to quantify the band density.

### 2.7. RNA Extraction and RT-qPCR

TRIzol reagent (Invitrogen) was used to extract the total cellular RNA. A 1 μg amount of total RNA was reverse transcribed to cDNA. The reverse transcription master kit (Takara, Kusatsu City, Japan) was used to synthesize the cDNA from the RNA. SYBR green (SYBR Green Supermix, Bio-Rad, Hercules, CA, USA) was used to perform the RT-qPCR analysis in a Bio-Rad CFX Real-Time PCR System (Bio-Rad). Samples were run in technical triplicates.

### 2.8. Xenograft Studies

For all animal studies, animals were randomly chosen, and no statistical method was used to predetermine sample size. The indicated A2780cisR cells (cell number 1 × 10^6^) were subcutaneously injected into the athymic nude mice (athymic nu/nu, female, 6-week-old, Charles River, Wilmington, MA, USA). The mice were evenly divided into each group for indicated treatment when the tumor size reached 100 mm^3^. For the treatment, mice were treated with cisplatin (5 mg/kg, IP, twice a week) and/or SP600125 (15 mg/kg, administered orally, daily) at a total daily dose from 3 days after xenograft inoculation until endpoint day. Tumor growth was recorded by blind measurement of two perpendicular diameters of the tumors, and tumor size was calculated using the formula 4π/3 × (width/2)2 × (length/2).

### 2.9. Statistical Analysis

Data from The Cancer Genome Atlas Ovarian Cancer (TCGA-OV) were analyzed by the GEPIA according to the instructions on the website [26]. The statistical analysis was performed with GraphPad Prism 8. No data were excluded. For sample size, no statistical method was used to predetermine sample size; a significant difference was detected in the preliminary studies in our assay, so we used the minimum sample size for all in vivo experiments. Data with error bars represent the mean ± standard deviation (SD). Values of *p* ≤ 0.05 were determined as statistically significant. Statistical analysis of significance was based on a two-tailed Student’s *t*-test or one-way or two-way ANOVA with Bonferroni post hoc multiple-comparisons testing as indicated. Statistical analyses are based on a set of assumptions, such as homogeneity of variances and normal distribution. Variance is similar between the groups that are being statistically compared. In vitro experiments were performed at least 3 times each, as is standard. Blinding was not performed in this study.

## 3. Results

### 3.1. XRCC4 Upregulation Is Associated with Cisplatin Resistance in Ovarian Cancer Cells

**Figure 1:**
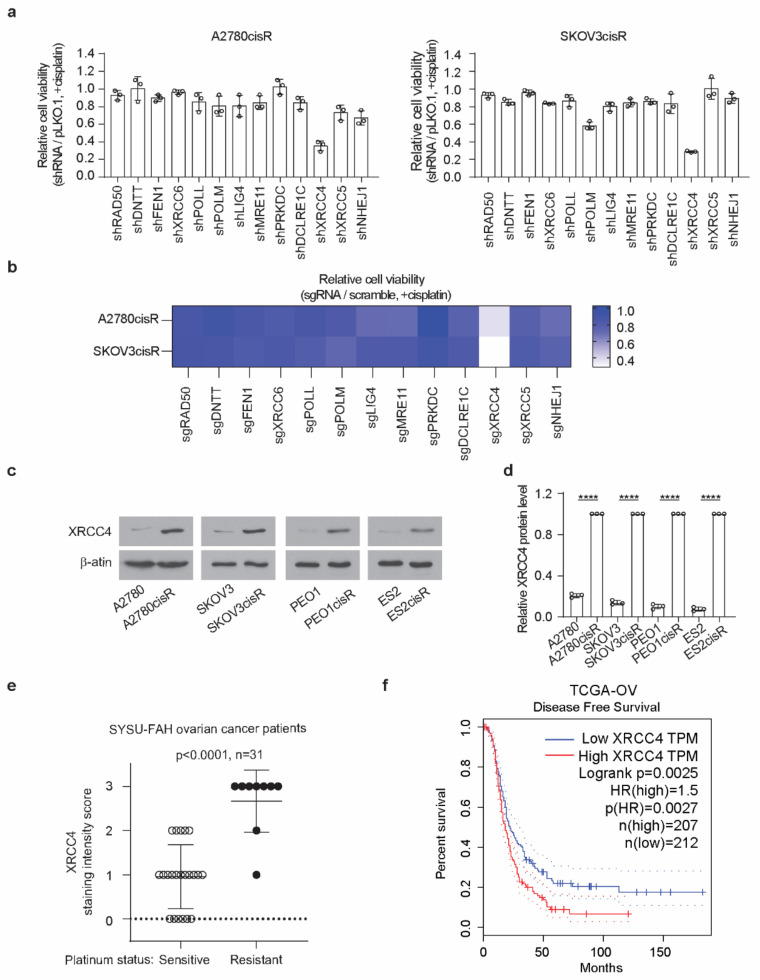
XRCC4 upregulation is associated with cisplatin resistance in ovarian cancer cells. (**a**) Results of synthetic lethality screen targeting 13 genes in NHEJ pathway using shRNAs with cisplatin in cisplatin-resistant ovarian cancer cell lines. A2780cisR and SKOV3cisR cells were infected with pooled shRNA lentivirus and sublethal doses of cisplatin (5 μg/mL A2780cisR and 2 μg/mL SKOV3cisR) for 48 h. Cell viability was determined by CellTiter-Glo luminescent cell viability assay. (**b**) Secondary screen used sgRNA to confirm the primary screen results. Cas9-expressing A2780cisR and SKOV3cisR cells were infected with sgRNA lentivirus and sublethal doses of cisplatin (5 μg/mL A2780cisR and 2 μg/mL SKOV3cisR) for 48 h. Cell viability was determined by CellTiter-Glo luminescent cell viability assay. (**c**) Representative XRCC4 expression level and (**d**) quantification in 4 pairs of cisplatin-resistant and parental ovarian cancer cells. The expression level was determined by Western blot and quantified by ImageJ. β-actin was used as loading control. (**e**) XRCC4 IHC staining score in the primary tumors from ovarian cancer patients who received platinum-based chemotherapy including cisplatin and carboplatin. Ovarian cancer patients were stratified into 2 groups: sensitive group (patients who responded to platinum-based chemotherapy durably for at least 6 months) and resistant group (patients who relapsed within the 6-month period and did not respond to platinum therapy). (**f**) Kaplan–Meier survival analysis of TCGA-OV cancer patients. Patients were dichotomized by XRCC4 expression level at median. Data are mean ± SD from 3 technical replicates of each sample and are representative of 2 (**a**), 2 (**b**), and 3 (**c**) independent biological experiments. Statistical analysis was performed by unpaired two-tailed *t*-test for (**b**,**d**). **** *p* < 0.0001.

**Figure A1:**
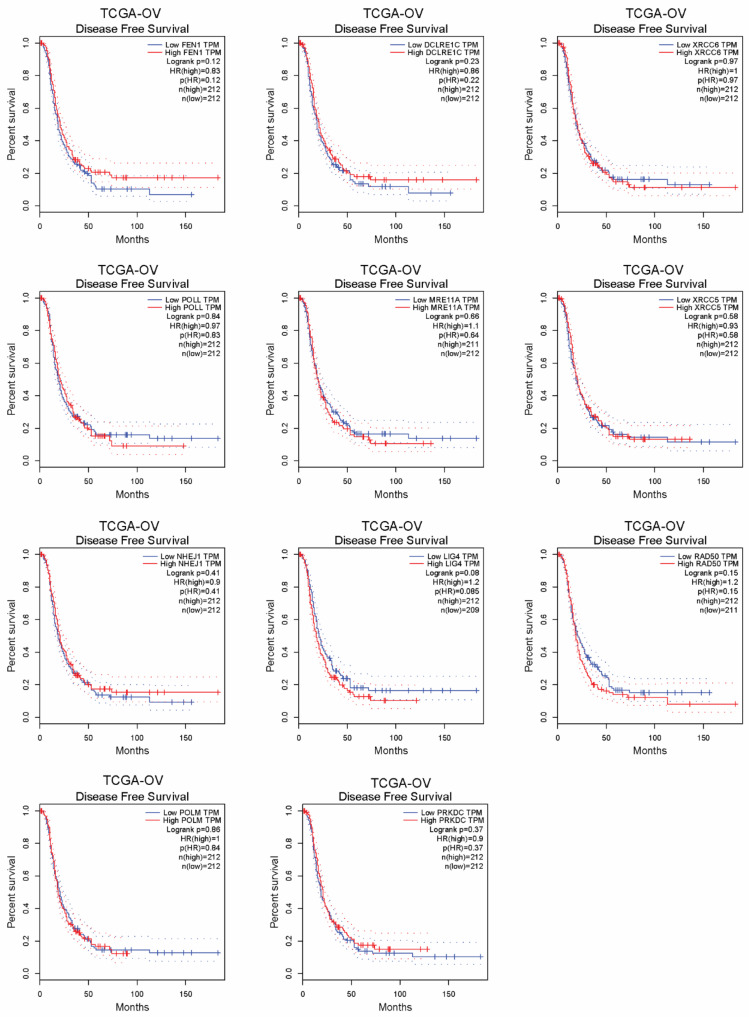
The correlation of genes in the NHEJ pathway with disease-free survival of ovarian cancer patients in TCGA database.

To better understand the link between the NHEJ pathway and its contribution to cisplatin resistance in ovarian cancer, we performed an NHEJ pathway-focused RNAi screen using a customized lentiviral short-hairpin RNA (shRNA) library. The screen individually targeted the 13 genes in the NHEJ pathway with a total of 64 shRNA constructs (Table S1). In the RNAi screen, 2 cisplatin-resistant cell lines, A2780cisR and SKOV3cisR, were transduced with the lentivirus pool containing shRNAs targeting each of the 13 individual genes before the treatment of the sublethal doses of cisplatin. Among the 13 genes examined, XRCC4 was identified as the most effective target that commonly and strongly sensitized the ovarian cancer cells to cisplatin treatment (Figure 1a). To exclude the possibility of an off-target effect by RNAi screen, a sgRNA-based screen was also performed. Cas9-expressing cell lines, A2780cisR and SKOV3cisR, were infected with the lentivirus pool containing sgRNAs targeting each of the 13 individual genes and treated with cisplatin (Supplementary Table S1). As shown in Figure 1b, XRCC4 loss attenuated the cell viability to the most in both cell lines upon cisplatin treatment, suggesting XRCC4 in the NHEJ pathway specifically plays an important role in conferring cisplatin resistance. Moreover, a strong increase in the XRCC4 expression was observed in a panel of four cisplatin-resistant ovarian cell lines relative to the paired parental cell lines (Figure 1c,d). Furthermore, XRCC4 expression and its relationship to cisplatin resistance were further examined in the primary ovarian cancer patient tumor who received platinum-based chemotherapy (Supplementary Table S2). Ovarian cancer patients were stratified into two groups: the sensitive group (*n* = 22, patients who responded to platinum-based chemotherapy durably for at least 6 months) and the resistant group (*n* = 9, patients who relapsed within the 6 months and did not respond to platinum therapy). A significantly higher expression level of XRCC4 was observed in the primary ovarian cancer tumor samples from the resistant group than from the sensitive group (Figure 1e). In addition, through the analysis of the TCGA-OV database, we also identify that high expression of XRCC4 significantly correlated with poor disease-free survival, implying XRCC4 expression possibly contributes to the resistance to platinum-based chemotherapy (Figure 1f). Intriguingly, the expression level of other genes in the NHEJ pathway did not exhibit a significant correlation with disease-free survival of ovarian cancer patients (Appendix A, Figure A1). Taken together, these data demonstrate that XRCC4 expression level positively correlates with cisplatin resistance in ovarian cancer cells.

### 3.2. Knockdown of XRCC4 Sensitized Cisplatin Treatment in the Cisplatin-Resistant Ovarian Cancer Cells

**Figure 2:**
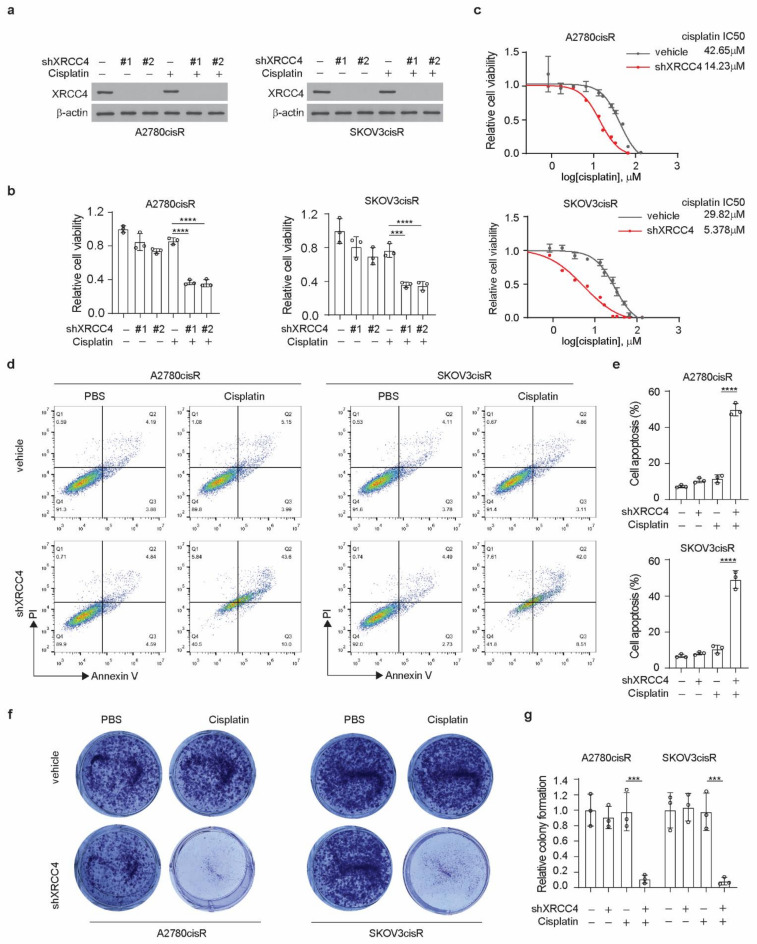
Knockdown of XRCC4 sensitized cisplatin treatment in the cisplatin-resistant ovarian cancer cells. (**a**) Confirmation of the knockdown of XRCC4 by 2 shRNAs in A2780cisR and SKOV3cisR treated with or without cisplatin by Western blot. (**b**) Cell viability of XRCC4 knockdown or vehicle control A2780cisR and SKOV3cisR cells treated with or without sublethal doses of cisplatin (5 μg/mL A2780cisR and 2 μg/mL SKOV3cisR) for 48 h. Cell viability was determined by CellTiter-Glo luminescent cell viability assay. (**c**) Cisplatin IC50 of A2780cisR and SKOV3cisR with or without XRCC4 knockdown. (**d**–**g**) Representative cell apoptosis (**d**), related quantification (**e**), representative colony formation potential (**f**), and related quantification (**g**) of A2780cisR and SKOV3cisR. Cells were transduced with XRCC4 shRNA clone and sublethal doses of cisplatin (5 μg/mL A2780cisR and 2 μg/mL SKOV3cisR) for 48 h. Data are mean ± SD from 3 technical replicates of each sample and are representative of 2 (**a**), 2 (**b**), 2 (**c**), 3 (**d**), and 2 (**f**) independent biological experiments. Statistical analysis was performed by 1-way ANOVA for (**b**,**d**,**e**); *** *p* < 0.001; **** *p* < 0.0001.

**Figure A2:**
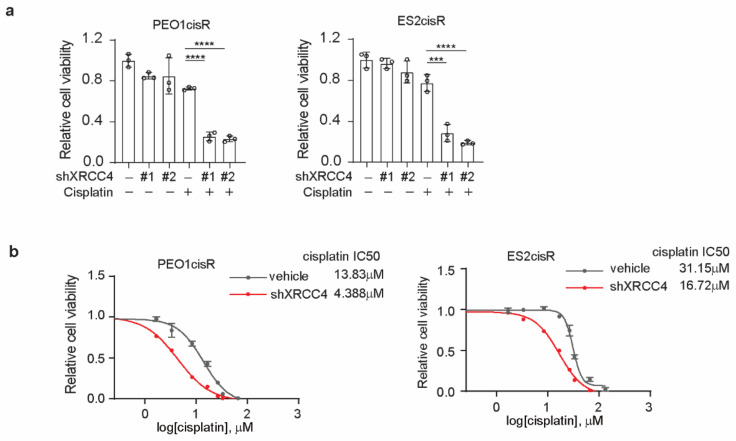
Knockdown of XRCC4 sensitized cisplatin treatment in the cisplatin-resistant ovarian cancer cells. (**a**) Cell viability of XRCC4 knockdown or vehicle control PEO1cisR and ES2cisR cells treated with or without sublethal doses of cisplatin (1.5 μg/mL PEO1cisR and 4 μg/mL ES2cisR) for 48 h. (**b**) Cisplatin IC50 of PEO1cisR and ES2cisR with or without XRCC4 knockdown. *** *p* < 0.001; **** *p* < 0.0001.

To study the role of XRCC4 in the response to cisplatin in ovarian cancer cells, we stably knocked down XRCC4 using two distinct shRNA clones in both the A2780cisR and SKOV3cisR cell lines. Successful knockdown of the XRCC4 was confirmed by the Western blot (Figure 2a). We observed that while the knockdown of XRCC4 alone did not significantly decrease the cell viability, the combination of XRCC4 knockdown and cisplatin treatment significantly attenuated the cell viability, suggesting the role of XRCC4 in sensitizing the cytotoxic effect of cisplatin in the ovarian cancer cells (Figure 2b). Consistent with the decreased cell viability, the IC50 of cisplatin in the XRCC4 knockdown cells was also significantly lower than in the control cells (Figure 2c). A similar pattern was also observed in ES2cisR and PEO1cisR upon XRCC4 knockdown (Figure A2a,b). Moreover, using FACS assay to monitor the cell apoptosis, we also found that the loss of XRCC4 robustly induced more cell death in the ovarian cancer cells treated with cisplatin (Figure 2d,e). A similar trend was also observed in the colony formation; the combination of XRCC4 knockdown and cisplatin treatment strongly abolished the colony formation potential in the cisplatin-resistant ovarian cancer cell lines, supporting the role of XRCC4 in the regulation of cisplatin sensitivity (Figure 2f,g). These data reveal that XRCC4 promotes cisplatin-resistant potential and that targeting XRCC4 sensitizes cisplatin-resistant ovarian cancer cells to cisplatin.

### 3.3. XRCC4 Loss Significantly Induced DNA Damage in Cisplatin-Resistant Ovarian Cells upon Cisplatin Treatment

**Figure 3:**
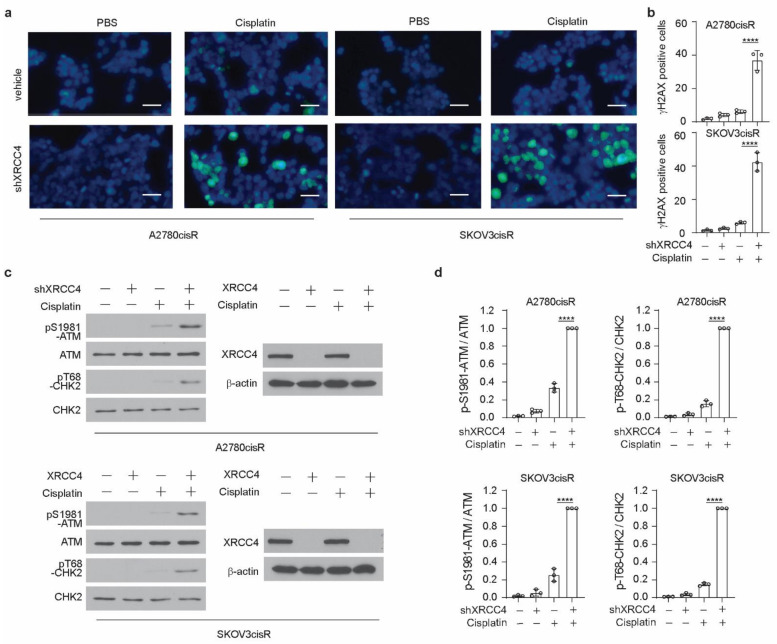
XRCC4 loss significantly induced DNA damage in cisplatin-resistant ovarian cells upon cisplatin treatment. (**a**,**b**) Representative immunofluorescence staining (**a**) of γH2AX (green) and DAPI (blue) and related quantification (**b**) in the indicated cells (treatment: cisplatin, 5 μg/mL A2780cisR, and 2 μg/mL SKOV3cisR for 48 h). Percentages of γH2AX-positive cells were quantified and are shown in the bar plots separately. Scale bar, 100 μm. (**c**,**d**) Representative blot (**c**) and quantification (**d**) of DNA damage checkpoint proteins in the indicated cells by Western blot (treatment: cisplatin, 5 μg/mL A2780cisR, and 2 μg/mL SKOV3cisR for 24 h). Indicated protein expression levels were quantified by ImageJ and are shown in the bar plots separately. Data are mean ± SD from 3 technical replicates of each sample and are representative of 2 (**a**) and 2 (**c**) independent biological experiments. Statistical analysis was performed by 1-way ANOVA for all data (**** *p* < 0.0001).

**Figure 4:**
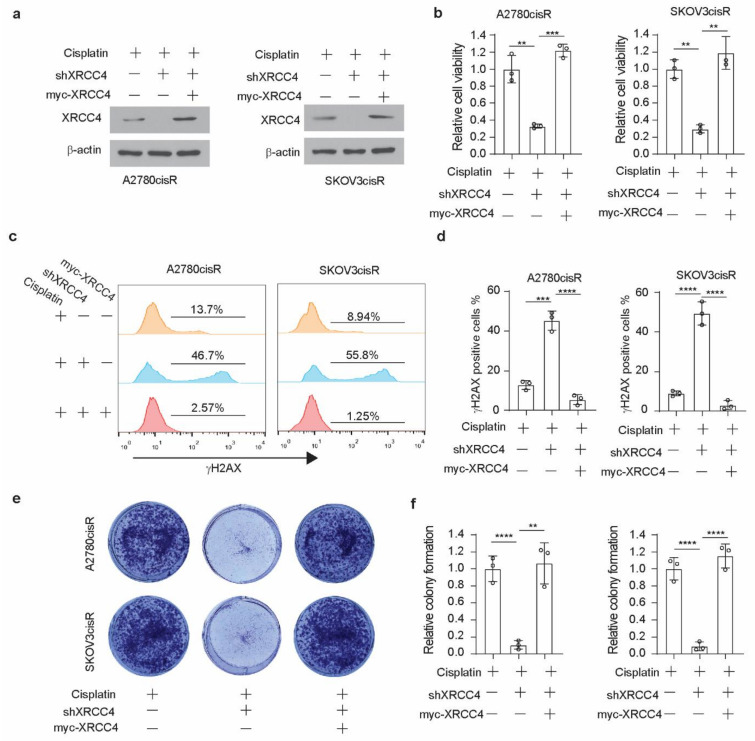
Rescued expression of XRCC4 reduced DNA damage in cisplatin-resistant ovarian cells upon cisplatin treatment. (**a**) Confirmation of the rescued expression of XRCC4 in the XRCC4 knockdown A2780cisR and SKOV3cisR by Western blot. (**b**) Cell viability of control, XRCC4 knockdown, and XRCC4 rescued A2780cisR and SKOV3cisR cells treated with the sublethal doses of cisplatin (5 μg/mL A2780cisR and 2 μg/mL SKOV3cisR) for 48 h. Cell viability was determined by CellTiter-Glo luminescent cell viability assay. (**c**,**d**) FACS analysis (**c**) and quantification (**d**) of γH2AX level in the indicated cells (treatment: cisplatin, 5 μg/mL A2780cisR, and 2 μg/mL SKOV3cisR for 48 h). (**e**,**f**) Colony formation potential (**e**) and quantification (**f**) of the indicated A2780cisR and SKOV3cisR. Cells were treated with the sublethal doses of cisplatin (5 μg/mL A2780cisR and 2 μg/mL SKOV3cisR) for 48 h. Data are mean ± SD from 3 technical replicates of each sample and are representative of 2 (**a**), 2 (**b**), and 3 (**c**–**e**) independent biological experiments. Statistical analysis was performed by 1-way ANOVA for all data; ** *p* < 0.01; *** *p* < 0.001; **** *p* < 0.0001.

XRCC4, a gene in the NHEJ pathway, is involved in the cellular response to DNA damage. Therefore, to glean insight into the mechanism by which XRCC4 sensitized cisplatin treatment, we first examined the level of DNA damage using the damage marker γH2AX. Surprisingly, through the immunofluorescence staining in both A2780cisR and SKOV3cisR cells, we observed that knockdown of XRCC4 led to a strong increase of the percentage of γH2AX positive cells under the treatment of cisplatin, suggesting that XRCC4 may involve in the regulation the response to the DNA damage caused by the cisplatin (Figure 3a,b). However, the excessive apoptosis with degradation of DNA will readily cause the detection of γH2AX by immunofluorescence. Nonetheless, we also used the Western blot to monitor the ATM-dependent signaling event profiling and found a consistently increased level of phosphorylation of ATM and CHK2 in the XRCC4 knockdown cells relative to control cells upon the treatment of cisplatin, further suggesting that XRCC4 knockdown sensitizes the cisplatin through aggravating the DNA damage (Figure 3c,d). To further confirm the role of XRCC4 in regulating cisplatin-induced DNA damage in ovarian cancer cells. We rescued the expression of XRCC4 in the A2780cisR and SKOV3cisR cells with endogenous XRCC4 knocked down. We confirmed the successfully rescued expression of XRCC4 by Western blot (Figure 4a). The rescued expression of XRCC4 significantly boosted the decreased cell viability caused by the combination of cisplatin treatment and XRCC4 knockdown (Figure 4b). Moreover, using FACS to monitor the DNA damage level through the analysis of γH2AX, we found that rescued the expression of XRCC4 robustly dampened the DNA damage conferred by the cisplatin in the XRCC4 knockdown cells (Figure 4c,d). Consistent with the increased cell viability and suppressed DNA damage, recused expression of XRCC4 resulted in a significant increase in the colony formation of the XRCC4 knockdown cells treated with cisplatin (Figure 4e,f). Taken together, the above results indicated that XRCC4 knockdown significantly increased the cisplatin sensitivity by promoting cisplatin-induced DNA damage in the cisplatin-resistant ovarian cancer cells.

### 3.4. cJUN Mediated the Upregulation of XRCC4 in Cisplatin-Resistant Ovarian Cells Treated with Cisplatin

**Figure 5:**
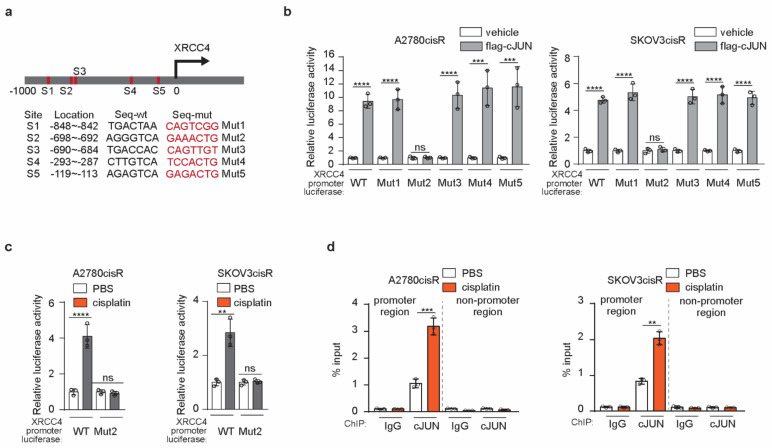
cJUN mediated the upregulation of XRCC4 in cisplatin-resistant ovarian cells treated with cisplatin (**a**) Schematic illustration of cJUN binding sites in the XRCC4 promoter. The wild-type (wt) and corresponding mutation sequences for each XRCC4 promoter-luciferase plasmids were provided (**b**) Analysis of the indicated XRCC4 promoter-luciferase activity in A2780cisR and SKOV3cisR cells transfected with control or cJUN overexpressing vector by luciferase activity assay. (**c**) Analysis of the indicated XRCC4 promoter-luciferase activity in A2780cisR and SKOV3cisR cells treated with PBS or cisplatin by luciferase activity assay. Treatment: cisplatin (5 μg/mL A2780cisR and 2 μg/mL SKOV3cisR) for 48 h (**d**) Analysis of cJUN binding to XRCC4 promoter region in A2780cisR and SKOV3cisR cells treated with PBS or cisplatin by ChIP. The IgG was used as control of cJUN antibody. The nonpromoter region of XRCC4 was also parallelly compared. Treatment: cisplatin (5 μg/mL A2780cisR and 2 μg/mL SKOV3cisR) for 48 h. Data are mean ± SD from 3 technical replicates of (**b**–**d**). Data are representative of 2 (**b**), 2 (**c**), and 2 (**d**), independent biological experiments. Statistical analysis was performed by 1-way ANOVA for all data; ns, not significant; ** *p* < 0.01; *** *p* < 0.001; **** *p* < 0.0001.

To glean a comprehensive mechanistic insight into how XRCC4 is upregulated to promote cisplatin-resistant in ovarian cancer cells, we first used the PROMO database to predict the transcription factors (TFs) that may mediate the transcription of XRCC4. cJUN among the predicted TFs was selected for further study. First of all, a total of five conserved sites for cJUN binding were observed in the XRCC4 promoter sequence (Figure 5a). Moreover, we previously reported that cJUN was specifically activated by cisplatin treatment in ovarian cancer cell lines. To further confirm that cJUN is the TF that drives the XRCC4 transcription upon cisplatin exposure, we generated the XRCC4 promoter-luciferase constructs with each individual predicted binding site mutated. As shown in Figure 5b, wild-type XRCC4 promoter-luciferase activity (WT) was significantly increased by cJUN overexpression in both the A2780cisR and SKOV3cisR cell lines, suggesting cJUN indeed promotes the expression of XRCC4 in ovarian cancer cells. Moreover, the specific mutation of binding site 2 abrogated the increased XRCC4 luciferase activity by the cJUN overexpression in both the A2780cisR and SKOV3cisR cell lines, suggesting that cJUN specifically binds to the S2 site (−698~−692) in the XRCC4 promoter sequence to drive its transcription. We further demonstrate that cJUN-mediated XRCC4 transcription is strongly boosted by cisplatin treatment. In both A2780cisR and SKOV3cisR cell lines, treatment of cisplatin led to a significant increase of XRCC4 promoter activity; by contrast, mutation of cJUN binding site to XRCC4 promoter diminished the effect of cisplatin in the upregulating of XRCC4 promoter activity. These data suggest that cisplatin promotes the transcription of XRCC4 through cJUN binding to the XRCC4 promoter sequence (Figure 5c). To further support the cJUN mediate the transcription of XRCC4 in ovarian cancer cells exposed to cisplatin, we perform a ChIP assay to monitor the endogenous binding of cJUN to XRCC4 promoter region in the chromosome in both A2780cisR and SKOV3cisR cell lines treated with cisplatin. As shown in Figure 5d, cisplatin treatment strongly promoted the specific physical interaction of the cJUN transcription factor and XRCC4 promoter region. Taken together, these data suggest that transcription factor cJUN promotes the upregulation of XRCC4 in cisplatin-resistant ovarian cells treated with cisplatin.

### 3.5. JNK-cJUN Axis Activated by Cisplatin Promotes XRCC4 Expression in the Cisplatin-Resistant Ovarian Cancer Cells

**Figure 6:**
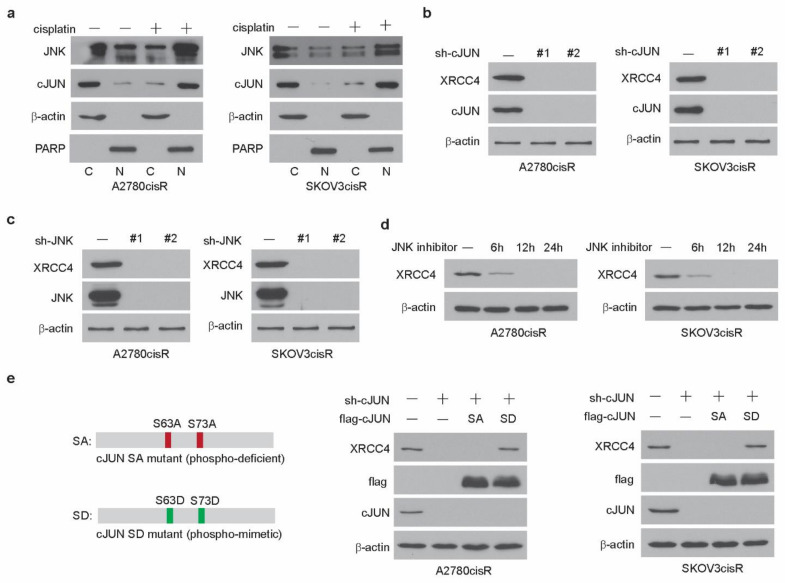
JNK-cJUN axis activated by cisplatin promotes XRCC4 expression in the cisplatin-resistant ovarian cancer cells (**a**) Localization of cJUN and JNK in cells treated with cisplatin. Treatment: cisplatin (5 μg/mL A2780cisR and 2 μg/mL SKOV3cisR) for 48 h. β-Actin and PARP were used as cytoplasmic and nuclear markers, respectively. C, cytosol, N, nucleus. (**b**,**c**) Detection of the level of XRCC4 by Western blot in A2780cisR and SKOV3cisR cells with/without the knockdown of cJUN (**b**) and JNK (**c**), respectively. Cells were treated with cisplatin. Treatment: cisplatin (5 μg/mL A2780cisR and 2 μg/mL SKOV3cisR) for 48 h. (**d**) Detection of the level of XRCC4 by Western blot in A2780cisR and SKOV3cisR cells treated with JNK inhibitor in a time-dependent manner. Cells were cotreated with cisplatin. Treatment: cisplatin (5 μg/mL A2780cisR and 2 μg/mL SKOV3cisR). (**e**) Left: Schematic illustration of cJUN SA (S63A/S73A) or SD (S63D/S73D) overexpression plasmid. Right: detection of the level of XRCC4 by Western blot in A2780cisR and SKOV3cisR cells with endogenous cJUN removed and rescued by cJUN SA or cJUN SA mutants. Data are representative of 2 (**a**), 3 (**b**), 2 (**c**), 3 (**d**), and 2 (**e**) independent biological experiments.

Our previous study demonstrated that DGKA activates the transcription factor cJUN in ovarian cancer cells upon cisplatin treatment [25]. Phosphorylation of cJUN at serine 63 by JNK is known to enhance the transcription activity of cJUN. Through the proteomic and genomic screens, we found that DGKA and its metabolic product PA enhanced the formation of cJUN and JNK complex, increased the cJUN phosphorylation level, and thus promoted their nuclear localization, which eventually leads to the high transcriptional activity of cJUN in ovarian cancer cells upon cisplatin exposure. In this study, we have shown that cJUN acted as the transcription factor of XRCC4 in the A2780cisR and SKOV3cisR treated with cisplatin. We then set out to examine how cJUN is activated to drive XRCC4 transcription. First, we observed that the complex of cJUN and JNK translocated into the nucleus in the cisplatin-resistant ovarian cancer cell lines exposed to cisplatin, suggesting that the JNK-cJUN axis was activated in the cisplatin-treated ovarian cancer cell lines (Figure 6a). More importantly, while the knockdown of cJUN abrogated the expression of XRCC4 (Figure 6b), JNK loss also completely abolished the expression of XRCC4 in cisplatin-resistant ovarian cancer cell lines (Figure 6c). Two individual shRNAs were used for these experiments to exclude the possibility of off-target. Moreover, as shown in Figure 6d, treatment of the JNK inhibitor decreased the expression level of XRCC4 in a time-point-dependent manner in the cisplatin-resistant ovarian cancer cell lines. These data suggest that the JNK-cJUN axis activated by cisplatin plays an important role in promoting the XRCC4 expression. After the formation of the complex, JNK phosphorylated the cJUN to promote its activation. To further prove that the phosphorylation of cJUN is important for its role in XRCC4 expression, we generated the cJUN mutant constructs, the constitutively active phospho-mimetic mutant form SD (S63D/S73D) and the constitutively inactive ¬¬ mutant form SA (S63A/S73A) of cJUN, respectively (Figure 6e). In the cJUN knockdown cell, rescued expression of the active SD mutant of cJUN but not the inactive SA mutant of cJUN restored the expression level of XRCC4 (Figure 6e). Taken together, these data suggest that the cisplatin-activated JNK-cJUN axis contributes to the upregulation of XRCC4 in the cisplatin-resistant ovarian cancer cells exposed to cisplatin.

### 3.6. Target Inhibition of JNK-cJUN-XRCC4 Pathway Sensitize Cisplatin-Resistant Ovarian Cancer to Cisplatin Treatment In Vitro and In Vivo

**Figure 7:**
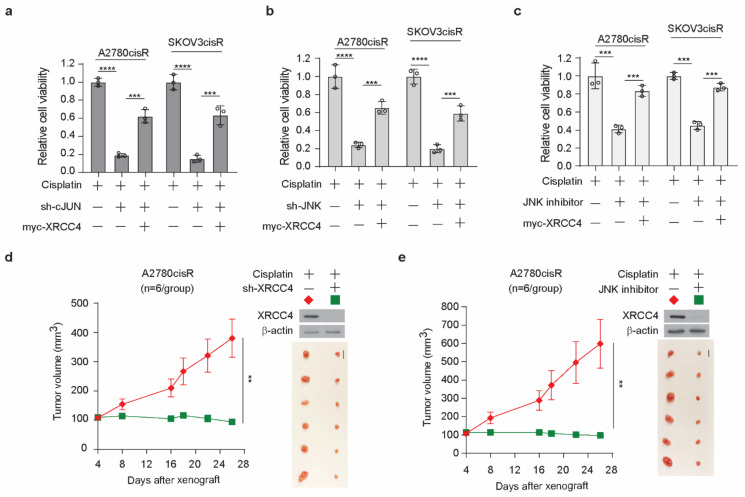
Target inhibition of JNK-cJUN-XRCC4 pathway sensitize cisplatin-resistant ovarian cancer to cisplatin treatment in vitro and in vivo (**a**,**b**) Cell viability of cisplatin-treated A2780cisR and SKOV3cisR cells with endogenous cJUN (**a**) or JNK (**b**) removed and XRCC4 overexpressed. (**c**) Cell viability of cisplatin-treated A2780cisR and SKOV3cisR cells with JNK inhibitor incubation and XRCC4 overexpressed. (**a**–**c**) Cells were all treated with the sublethal doses of cisplatin (5 μg/mL A2780cisR and 2 μg/mL SKOV3cisR) for 48 h. Cell viability was determined by CellTiter-Glo luminescent cell viability assay. (**d**,**e**) Effect of XRCC4 knockdown or pharmaceutical inhibition of JNK on the cisplatin-treated tumor growth of ovarian cancer cell line A2780cisR xenograft model. Mice were treated with cisplatin (5 mg/kg) by i.p. injection twice a week. JNK inhibitor SP600125 was administered orally at a total daily dose of 15 mg/kg. Tumor volume and dissected tumors are shown. Scale bar represents 1 cm for dissected tumors. Data are representative of 2 (**a**), 2 (**b**), and 2 (**c**) independent biological experiments. Data are shown as means ± SD (For statistical analyses: tumor volume: 2-way ANOVA; other assay: 1-way ANOVA); ** *p* < 0.01; *** *p* < 0.001; **** *p* < 0.0001.

We demonstrated that the knockdown of XRCC4 sensitized cisplatin treatment and the JNK-cJUN axis promotes the upregulation of XRCC4 to confer cisplatin resistance in ovarian cancer cells. Targeting the JNK-cJUN-XRCC4 would presumably hold the potential clinical translation of overcoming cisplatin resistance in ovarian cancer. However, the specific inhibitor of XRCC4 is not commercially available to our knowledge; the possible target strategy could be the inhibition of the JNK-cJUN pathway to control the level of XRCC4. First, we observed that knockdown of cJUN or JNK indeed significantly sensitized cisplatin treatment and forced overexpression of XRCC4 partially restored the cell viability, suggesting XRCC4 acted downstream of JNK-cJUN axis to promote cisplatin resistance (Figure 7a,b). Meanwhile, forced expression of XRCC4 also reduced the cisplatin-sensitizing effect of the JNK inhibitor (Figure 7c), further supporting that the pharmaceutical inhibition of JNK increased cisplatin sensitivity at least partly through the suppression of the XRCC4 expression. Next, we examined the effect of inhibition of the JNK-cJUN-XRCC4 pathway in sensitizing cisplatin treatment in vivo using the A2780cisR cell line xenograft mouse model. As shown in Figure 7d, the knockdown of XRCC4 significantly attenuated the tumor growth relative to the control under the treatment of cisplatin, suggesting XRCC4 loss sensitizing cisplatin treatment in vivo. More importantly, we also found that pharmaceutical inhibition of JNK significantly led to the downregulation of XRCC4 in the tumor cells and suppressed the tumor growth in the in vivo model (Figure 7e), indicating the important role of JNK-cJUN-XRCC4 in the response of cisplatin-resistant ovarian cancer cells to cisplatin treatment. These results strongly support that JNK-cJUN-XRCC4 could be targeted to overcome cisplatin resistance in ovarian cancer.

## 4. Discussion

**Figure A3:**
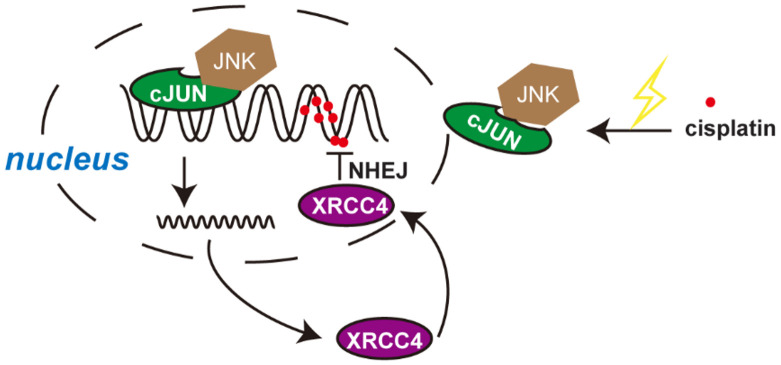
Illustration of working model. Cisplatin treatment induced the activation of the JNK-cJUN axis, which translocated to the nucleus and drive the transcription of XRCC4. XRCC4 contributes to DNA repair through the NHEJ pathway and therefore promotes cisplatin resistance in ovarian cancer.

Here we identify XRCC4 as a critical factor to confer cisplatin resistance in ovarian cancer. Through an NHEJ-pathway-focused RNAi screen, we found that XRCC4 specifically sensitized cisplatin treatment in cisplatin-resistant ovarian cancer cell lines, which were further confirmed by a sgRNA-based secondary screen. XRCC4 expression significantly increased in a variety of ovarian cancer cell lines when getting resistant to cisplatin. We further observed that XRCC4 loss significantly attenuated cell viability, cisplatin IC50, cell apoptosis, and colony formation in the cisplatin-resistant cell lines treated with cisplatin, confirming the XRCC4 plays an important role in cisplatin resistance in ovarian cancer. We further dissected the mechanism underlying the role of XRCC4 in the regulation of cisplatin sensitivity. XRCC4 knockdown was found to strongly aggravate the DNA damage caused by cisplatin treatment. Rescued expression of XRCC4 in the XRCC4 knockdown cells significantly reduced the cisplatin-induced DNA damage and restored the resistance to cisplatin in the ovarian cancer cell lines. Moreover, we found that the JNK-cJUN axis mediated the transcription and upregulation of XRCC4 in ovarian cancer cells exposed to cisplatin. Cisplatin treatment induced the activation of the JNK-cJUN axis, which translocated to the nucleus and drove the transcription of XRCC4 (Figure A3). Therefore, the cJUN-JNK-XRCC4 pathway contributes to cisplatin resistance in ovarian cancer.

Platinum-based chemotherapy has been the cornerstone for the clinical treatment of multiple cancer types. Many studies have revealed extensively the mechanisms that contribute to cisplatin resistance in cancer cells. Our previous research has demonstrated that kinase signaling networks play an important role in regulating the cellular response to cisplatin in ovarian cancer [9,10,24,25]. It is well known that the DNA damage response and repair pathway is deeply intertwined with the kinase network. The kinases ATM and ATR are the main factors orchestrating the DNA damage response and homologous recombination [27]. In the current study, we demonstrated that XRCC4-mediated NHEJ DNA damage repair also contributes to cisplatin resistance in ovarian cancer. Interestingly, our study also revealed that the expression of XRCC4 is controlled by the kinase JNK, reminiscent of the kinases we have identified to play critical roles in conferring cisplatin resistance in previous studies [25]. Nonetheless, the dissection of the mechanism in the current study relied mainly on two cell lines. More cell lines and human samples could be included in future studies to further validate the role of XRCC4 in conferring resistance to cisplatin. Notably, these studies indicated that cisplatin resistance is a systematic cellular response, necessitating a complex and complementary array of mechanisms, including cell survival signaling, cell metabolism, and DNA damage response, to work together against cisplatin.

The current study demonstrated the cisplatin treatment activated the JNK-cJUN complex to drive the transcription of XRCC4 yet did not further explore how the JNK-cJUN complex was activated as such. In our previous study, we found that in response to cisplatin, PA, the metabolite of diacylglycerol kinase alpha DGKA, directly promoted the formation and thus activation of the JNK and cJUN complex in the ovarian cancer cells. So, the JNK-cJUN complex that mediated the transcription of XRCC4 was also possibly promoted by DGKA in the ovarian cancer cells treated with cisplatin. Moreover, in our previous study, we found that the DGKA signals through JNK-cJUN drive the transcription of WEE1, a kinase involved in DNA damage checking point response 26. The current study showed that XRCC4 is possibly also controlled by the DGKA-JNK-cJUN pathway. These studies strengthened the central role of the DGKA-JNK-cJUN axis in the regulation of DNA damage repair in cisplatin-resistant ovarian cancer cells.

Clinically, our findings support that XRCC4 could serve as a predictive marker and as a promising therapeutic target to treat cisplatin-resistant ovarian cancer. Our data showed that XRCC4 level is significantly correlated with PFS in ovarian cancer patients, and targeting XRCC4 or the upstream kinase JNK strongly attenuated the cisplatin-resistant tumor growth of ovarian cancer cells in the preclinical mouse model. Future studies are warranted to develop and apply more specific and effective XRCC4 inhibitors to overcome cisplatin resistance in the clinic.

## 5. Conclusions

Our study reveals that the XRCC4 in the NHEJ pathway contributes to platinum resistance by reducing DNA damage and provides preclinical evidence for targeting XRCC4 as a new strategy to overcome cisplatin resistance in ovarian cancer treatment.

## Data Availability

No new data were created or analyzed in this study. Data sharing is not applicable to this article.

## Supplementary Materials

The following supporting information can be downloaded at: https://www.mdpi.com/article/10.3390/cancers14246068/s1, Table S1: shRNA and sgRNA sequences; Table S2: patient information.

## References

[1] Siegel R.L., Miller K.D., Fuchs H.E., Jemal A. (2022). Cancer statistics, 2022. CA Cancer J. Clin..

[2] Lawton F.G., Pavlik E.J. (2022). Perspectives on Ovarian Cancer 1809 to 2022 and Beyond. Diagnostics.

[3] Zhu S., Zhang C., Cao D., Bai J., Yu S., Chen J., Wang J., Ren T., Yang J., Yu M. (2022). Genomic and TCR profiling data reveal the distinct molecular traits in epithelial ovarian cancer histotypes. Oncogene.

[4] Ozols R.F. (1991). Ovarian cancer: New clinical approaches. Cancer Treat. Rev..

[5] Jackson S.P., Helleday T. (2016). DNA REPAIR. Drugging DNA repair. Science.

[6] Hong Z., Zhang W., Ding D., Huang Z., Yan Y., Cao W., Pan Y., Hou X., Weroha S.J., Karnes R.J. (2020). DNA Damage Promotes TMPRSS2-ERG Oncoprotein Destruction and Prostate Cancer Suppression via Signaling Converged by GSK3beta and WEE1. Mol. Cell.

[7] Galluzzi L., Senovilla L., Vitale I., Michels J., Martins I., Kepp O., Castedo M., Kroemer G. (2012). Molecular mechanisms of cisplatin resistance. Oncogene.

[8] Ishida S., McCormick F., Smith-McCune K., Hanahan D. (2010). Enhancing tumor-specific uptake of the anticancer drug cisplatin with a copper chelator. Cancer Cell.

[9] Pan C., Chun J., Li D., Boese A.C., Li J., Kang J., Umano A., Jiang Y., Song L., Magliocca K.R. (2019). Hsp90B enhances MAST1-mediated cisplatin resistance by protecting MAST1 from proteosomal degradation. J. Clin. Investig..

[10] Li J., Zheng C., Wang M., Umano A.D., Dai Q., Zhang C., Huang H., Yang Q., Yang X., Lu J. (2022). ROS-regulated phosphorylation of ITPKB by CAMK2G drives cisplatin resistance in ovarian cancer. Oncogene.

[11] Jin L., Chun J., Pan C., Li D., Lin R., Alesi G.N., Wang X., Kang H.B., Song L., Wang D. (2018). MAST1 Drives Cisplatin Resistance in Human Cancers by Rewiring cRaf-Independent MEK Activation. Cancer Cell.

[12] Liu D., Zhang X.X., Li M.C., Cao C.H., Wan D.Y., Xi B.X., Tan J.H., Wang J., Yang Z.Y., Feng X.X. (2018). C/EBPbeta enhances platinum resistance of ovarian cancer cells by reprogramming H3K79 methylation. Nat. Commun..

[13] Damia G., Broggini M. (2019). Platinum Resistance in Ovarian Cancer: Role of DNA Repair. Cancers.

[14] Da Cunha Colombo Bonadio R.R., Fogace R.N., Miranda V.C., Diz M. (2018). Homologous recombination deficiency in ovarian cancer: A review of its epidemiology and management. Clinics.

[15] Mehta A.K., Cheney E.M., Hartl C.A., Pantelidou C., Oliwa M., Castrillon J.A., Lin J.R., Hurst K.E., de Oliveira Taveira M., Johnson N.T. (2021). Targeting immunosuppressive macrophages overcomes PARP inhibitor resistance in BRCA1-associated triple-negative breast cancer. Nat. Cancer.

[16] Biegala L., Gajek A., Marczak A., Rogalska A. (2021). PARP inhibitor resistance in ovarian cancer: Underlying mechanisms and therapeutic approaches targeting the ATR/CHK1 pathway. Biochim. Biophys. Acta Rev. Cancer.

[17] Chen X., Xu X., Chen Y., Cheung J.C., Wang H., Jiang J., de Val N., Fox T., Gellert M., Yang W. (2021). Structure of an activated DNA-PK and its implications for NHEJ. Mol. Cell.

[18] Chen S., Lee L., Naila T., Fishbain S., Wang A., Tomkinson A.E., Lees-Miller S.P., He Y. (2021). Structural basis of long-range to short-range synaptic transition in NHEJ. Nature.

[19] Chaplin A.K., Hardwick S.W., Stavridi A.K., Buehl C.J., Goff N.J., Ropars V., Liang S., de Oliveira T.M., Chirgadze D.Y., Meek K. (2021). Cryo-EM of NHEJ supercomplexes provides insights into DNA repair. Mol. Cell.

[20] Yang Y., Yang C., Li T., Yu S., Gan T., Hu J., Cui J., Zheng X. (2020). The Deubiquitinase USP38 Promotes NHEJ Repair through Regulation of HDAC1 Activity and Regulates Cancer Cell Response to Genotoxic Insults. Cancer Res..

[21] Wang H., Perrault A.R., Takeda Y., Qin W., Wang H., Iliakis G. (2020). Biochemical evidence for Ku-independent backup pathways of NHEJ. Nucleic Acids Res..

[22] Patterson-Fortin J., Bose A., Tsai W.C., Grochala C., Nguyen H., Zhou J., Parmar K., Lazaro J.B., Liu J., McQueen K. (2022). Targeting DNA Repair with Combined Inhibition of NHEJ and MMEJ Induces Synthetic Lethality in TP53-Mutant Cancers. Cancer Res..

[23] Liang L., Feng J., Zuo P., Yang J., Lu Y., Yin Y. (2020). Molecular basis for assembly of the shieldin complex and its implications for NHEJ. Nat. Commun..

[24] Pan C., Jin L., Wang X., Li Y., Chun J., Boese A.C., Li D., Kang H.B., Zhang G., Zhou L. (2019). Inositol-triphosphate 3-kinase B confers cisplatin resistance by regulating NOX4-dependent redox balance. J. Clin. Investig..

[25] Li J., Pan C., Boese A.C., Kang J., Umano A.D., Magliocca K.R., Yang W., Zhang Y., Lonial S., Jin L. (2020). DGKA Provides Platinum Resistance in Ovarian Cancer through Activation of c-JUN-WEE1 Signaling. Clin. Cancer Res..

[26] Tang Z., Li C., Kang B., Gao G., Li C., Zhang Z. (2017). GEPIA: A web server for cancer and normal gene expression profiling and interactive analyses. Nucleic Acids Res..

[27] Marechal A., Zou L. (2013). DNA damage sensing by the ATM and ATR kinases. Cold Spring Harb. Perspect. Biol..

